# Effectiveness of dry needling combined with exercise therapy versus exercise alone for pain and functional outcomes in knee osteoarthritis: a systematic review and meta-analysis of randomized controlled trials

**DOI:** 10.1177/1759720X261463350

**Published:** 2026-07-13

**Authors:** Muhammad Tayyab, Ameer Afzal Khan, Rahman Syed, Suleman Shah, Mohammed Al Meqbaali, Anfal Khan, Mohammed Al Sinani

**Affiliations:** Department of Orthopedics and Trauma, Bradford Teaching Hospitals NHS Foundation Trust, Bradford, England, UK; Department of Internal Medicine, Saidu Medical College, Saidu Sharif, Pakistan; Department of Internal Medicine, Swat Medical College, Saidu Sharif, Pakistan; Department of Nursing, Fatima College of Health Sciences—Al Ain Campus, Al Ain, Abu Dhabi, United Arab Emirates; Department of Nursing, Fatima College of Health Sciences—Al Ain Campus, Al Ain, Abu Dhabi, United Arab Emirates; Department of Internal Medicine, Saidu Medical College, Saidu Sharif, Pakistan; Department of Metabolism, Digestion and Reproduction, Imperial College London, 369 Fulham Road, London SW10 9NH, UK

**Keywords:** dry needling, exercise therapy, knee osteoarthritis, pain, WOMAC

## Abstract

**Background:**

Knee osteoarthritis (KOA) is a leading cause of chronic pain and disability worldwide. Although exercise therapy is the recommended first-line treatment, many patients continue to experience pain and functional limitations. Dry needling (DN) has emerged as a potential adjunct therapy targeting myofascial and neuropathic pain mechanisms.

**Objectives:**

To evaluate the effectiveness of DN combined with exercise therapy compared with exercise therapy alone for pain reduction and functional improvement in patients with KOA.

**Design:**

Systematic review and meta-analysis of randomized controlled trials.

**Data sources and methods:**

A systematic search of PubMed, ClinicalTrials.gov, and the Cochrane Library was conducted from inception to October 23, 2025, following Preferred Reporting Items for Systematic Reviews and Meta-Analyses 2020 guidelines. Studies involving adults with KOA comparing DN plus exercise with exercise alone or sham DN plus exercise were included. The primary outcome was pain intensity (Visual Analog Scale/Numeric Pain Rating Scale), while secondary outcomes included WOMAC scores, neuropathic pain, functional performance, and psychosocial measures. Pooled mean differences (MDs) with 95% confidence intervals (CIs) were calculated using a random-effects model. Heterogeneity was assessed with the *I*^2^ statistic, and risk of bias was evaluated using the Cochrane RoB-2 tool.

**Results:**

Six randomized controlled trials involving 453 participants were included. Compared with exercise alone, DN plus exercise significantly reduced pain at 3 months (MD −1.91; 95% CI −2.60 to −1.21; *I*^2^ = 66%). Significant improvements were also observed in WOMAC pain, stiffness, and physical function at short- and mid-term follow-up. Long-term outcomes (6–12 months) showed favorable trends, although CIs were wider.

**Conclusion:**

DN combined with exercise therapy may offer short- to mid-term improvements in pain and function in patients with KOA compared with exercise alone. However, the evidence is limited by a small number of studies, moderate certainty, and substantial heterogeneity, warranting cautious interpretation. The long-term benefits remain uncertain.

***Trial registration*:** PROSPERO (Registration ID: (CRD420261285294)).

## Plain language summary

Does adding dry needling to exercise help reduce pain and improve movement in people with knee arthritis?

Knee osteoarthritis is a common condition that causes long-term knee pain, stiffness, and difficulty walking or doing daily activities. Exercise is one of the most recommended treatments because it helps strengthen the muscles around the knee and improve movement. However, many people still have ongoing pain even after following an exercise program. Dry needling is a treatment where a trained healthcare professional inserts thin needles into tight or painful muscle areas. It is different from acupuncture and is used to reduce muscle tension and pain. Some therapists use dry needling together with exercise, but it is not clear whether this combination works better than exercise alone. In this study, we reviewed and combined results from six high-quality clinical trials including 453 people with knee osteoarthritis. We compared people who received dry needling plus exercise with those who received exercise alone. Our findings showed that people who received dry needling together with exercise had greater pain relief and better physical function in the short and medium term (around 3 months after treatment). Improvements in stiffness and daily activities were also seen. The long-term benefits were less certain, as fewer studies followed patients for longer periods. Overall, adding dry needling to an exercise program may provide extra short-term benefits for people with knee osteoarthritis. More research is needed to understand how long these benefits last.

## Introduction

Knee osteoarthritis (KOA) is one of the most common chronic musculoskeletal disorders in the world, and it is a major cause of pain, disability, and poor quality of life in older adults.^1^ It is characterized by articular cartilage degeneration, subchondral bone remodeling, synovial inflammation, and periarticular muscle dysfunction.^2^ The global burden of KOA is increasing due to population aging, obesity, and sedentary lifestyles, which contribute significantly to healthcare costs and functional impairment.^3^ Patients frequently report chronic pain, stiffness, limited mobility, and reduced participation in daily and social activities.^4^

Current clinical guidelines consistently recommend exercise therapy as the primary non-pharmacological treatment for KOA.^5^ Structured exercise programs increase muscle strength, joint stability, and functional capacity while decreasing pain and impairment.^6^ Despite its well-established benefits, a significant minority of patients continue to experience prolonged pain and inadequate functional improvement after exercising alone.^7^ These findings indicate that additional pain-relieving therapies may be required to optimize therapeutic outcomes.

Recent research suggests that myofascial trigger points and peripheral sensitization play a role in the pathogenesis of osteoarthritis pain.^8^ Dry needling (DN), a minimally invasive procedure for treating myofascial trigger points, has gained popularity as an adjuvant intervention in musculoskeletal problems. DN is thought to diminish nociceptive input, enhance local circulation, reduce muscle tension, and influence central pain pathways.^9^ Electrical dry needling (EDN), which combines needling and electrical stimulation, has the potential to boost neuromodulatory effects.^10^

While previous systematic reviews have examined DN as a standalone or combined intervention for KOA,^11–13^ these reviews focused primarily on DN versus passive controls or other modalities rather than performing a focused synthesis comparing “dry needling plus exercise” versus “exercise alone”—the most clinically relevant comparison for rehabilitation practice. Specifically, Jiménez-del-Barrio et al.,^11^ Rahou-El-Bachiri et al.,^12^ and Ughreja and Prem^13^ each examined DN as a standalone treatment or versus passive controls, and none performed a quantitative synthesis of the additive benefit of DN when combined with structured exercise therapy as the primary comparator. Our review specifically addresses this evidence gap.

Several RCTs have investigated the effects of DN combined with exercise therapy in KOA.^14–18^ However, findings vary in terms of intervention methods, follow-up duration, and reported effects. Given the growing clinical use of DN in rehabilitation settings, a focused systematic review and meta-analysis is required to synthesize this evidence. The objective of this study was to compare the effectiveness of DN combined with exercise therapy to exercise therapy alone for pain relief and functional outcomes in patients with KOA.

## Methods

This meta-analysis was conducted in accordance with the Preferred Reporting Items for Systematic Reviews and Meta-Analyses guidelines. The study adhered to the principles of the Declaration of Helsinki and was exempt from Institutional Review Board approval as it involved secondary analysis of previously published studies. The review protocol was registered in the International Prospective Register of Systematic Reviews (PROSPERO; ID: (CRD420261285294)).

### Information sources

A comprehensive literature search was performed across PubMed (NCBI), ClinicalTrials.gov, and the Cochrane Library from database inception to October 23, 2025, without language restrictions.

### Search strategy

The search strategy incorporated both Medical Subject Headings (MeSH) and free-text terms related to DN, exercise therapy, and KOA. The complete PubMed search string, including MeSH terms and field tags, was:

(“osteoarthritis, knee”[MeSH Terms] OR (“osteoarthritis, knee”[MeSH Terms] OR (“osteoarthritis”[All Fields] AND “knee”[All Fields]) OR “knee osteoarthritis”[All Fields] OR (“knee”[All Fields] AND “osteoarthritides”[All Fields]) OR “knee osteoarthritides”[All Fields]) OR (“osteoarthritis, knee”[MeSH Terms] OR (“osteoarthritis”[All Fields] AND “knee”[All Fields]) OR “knee osteoarthritis”[All Fields] OR (“knee”[All Fields] AND “osteoarthritis”[All Fields])) OR (“osteoarthritis, knee”[MeSH Terms] OR (“osteoarthritis”[All Fields] AND “knee”[All Fields]) OR “knee osteoarthritis”[All Fields] OR “osteoarthritis of knee”[All Fields])) AND (“Dry Needling”[MeSH Terms] OR (“Dry Needling”[MeSH Terms] OR (“dry”[All Fields] AND “needling”[All Fields]) OR “Dry Needling”[All Fields] OR (“needling”[All Fields] AND “dry”[All Fields]) OR “needling dry”[All Fields])) AND (“Exercise”[MeSH Terms] OR (“Exercise”[MeSH Terms] OR “Exercise”[All Fields] OR “exercises”[All Fields] OR “exercise therapy”[MeSH Terms] OR (“Exercise”[All Fields] AND “therapy”[All Fields]) OR “exercise therapy”[All Fields] OR “exercising”[All Fields] OR “exercise s”[All Fields] OR “exercised”[All Fields] OR “exerciser”[All Fields] OR “exercisers”[All Fields]) OR (“Exercise”[MeSH Terms] OR “Exercise”[All Fields] OR (“Exercise”[All Fields] AND “physical”[All Fields]) OR “exercise physical”[All Fields]) OR (“Exercise”[MeSH Terms] OR “Exercise”[All Fields] OR (“physical”[All Fields] AND “Exercise”[All Fields]) OR “physical exercise”[All Fields]) OR (“Exercise”[MeSH Terms] OR “Exercise”[All Fields] OR (“Exercise”[All Fields] AND “isometric”[All Fields]) OR “exercise isometric”[All Fields]) OR (“Exercise”[MeSH Terms] OR “Exercise”[All Fields] OR (“Exercise”[All Fields] AND “training”[All Fields]) OR “exercise training”[All Fields]))

The ClinicalTrials.gov search was conducted using the following terms entered into the Condition or Disease field and Intervention/Treatment field respectively: “knee osteoarthritis” AND “dry needling” AND “exercise,” filtered to Interventional Studies (Clinical Trials) only. No date or status filters were applied.

The Cochrane Library (CENTRAL) search strategy was structured as follows. First, KOA was captured by combining a MeSH descriptor search ([Osteoarthritis, Knee] explode all trees) with a free-text search for “knee osteoarthritis,” “knee OA,” and “gonarthrosis” across title, abstract, and keyword fields, and these two sets were combined using OR. Second, DN was identified by combining a MeSH descriptor search ([Dry Needling] explode all trees) with a free-text search for “dry needling,” “intramuscular stimulation,” and “electrical dry needling” across title, abstract, and keyword fields, again combined using OR. Third, exercise therapy was captured by combining a MeSH descriptor search ([Exercise Therapy] explode all trees) with a free-text search for “exercise,” “physiotherapy,” “rehabilitation,” and “manual therapy” across title, abstract, and keyword fields, combined using OR. The three concept sets were then combined using AND to retrieve records relevant to all three domains simultaneously. No language restrictions or date limits were applied to this search, which was conducted on October 23, 2025.

Additional searches included combinations such as “dry needling plus exercise,” “sham dry needling,” and “electrical dry needling” to identify relevant randomized trials. Reference lists of included studies and prior systematic reviews were manually screened to identify additional eligible trials. Duplicate records were removed prior to screening.

### Study selection

Two reviewers independently screened titles and abstracts for eligibility, followed by full-text assessment of potentially relevant articles. Discrepancies were resolved through discussion or consultation with a third reviewer.

Studies were included if they met all of the following criteria: (1) Randomized controlled trials (RCTs) with a parallel-group design (non-crossover). (2) Adult participants (⩾18 years) diagnosed clinically or radiographically with KOA (any Kellgren–Lawrence grade), in outpatient or clinical rehabilitation settings. (3) The intervention group received DN in combination with exercise therapy, with or without manual therapy. (4) Comparator group received exercise therapy alone or sham DN plus exercise. (5) Reported at least one quantitative outcome related to pain (Visual Analog Scale (VAS), Numeric Pain Rating Scale (NPRS)) or function (WOMAC, KOOS, lower extremity function (LEFS)). (6) Reported mean ± standard deviation (SD) or sufficient data for effect-size calculation. (7) Published as full-text articles in peer-reviewed journals.

Exclusion criteria were: (1) non-randomized or observational studies, (2) Animal or pediatric studies, (3) Conference abstracts without full data, (4) Studies lacking a clear comparator group, (5) Trials evaluating DN without an exercise component, (6) Studies where outcomes of interest (pain on VAS/NPRS or function on WOMAC/KOOS/LEFS) were not measured or not reported in a format compatible with meta-analytic pooling.

No restrictions were placed on year of publication or language of the report. No minimum follow-up duration was mandated; studies reporting at least one post-intervention outcome time point were eligible.

### Data extraction

Data extraction was independently performed by two reviewers using a standardized data extraction form. Extracted variables included author and year, study design, sample size, participant characteristics, intervention and comparator details (including DN type, frequency, and exercise protocol), follow-up duration, and reported outcomes.

Primary and secondary outcome data were extracted as post-intervention values and follow-up means with corresponding SD. When required numerical data were missing or unclear, attempts were made to contact the study authors. Any disagreements were resolved through consensus or third-reviewer adjudication.

For each included study, data were sought for all results compatible with each pre-specified outcome domain, across all measurement instruments, time points, and analyses reported. Where a study reported pain intensity using both VAS and NPRS, both were recorded; for pooling, the instrument more consistently reported across studies was prioritized, and where scales were compatible (e.g., 0–10 point scales), data were combined. Outcomes were extracted at all available follow-up time points (post-intervention (0–2), 3, 6, and 12 months). Where a study reported data only at a time point not pre-specified in the analysis plan, results were reported narratively. The selection of extracted results for each synthesis was based on pre-specified outcome domains and follow-up subgroups defined a priori in the registered protocol (PROSPERO ID: CRD420261285294).

In addition to primary and secondary outcome data, the following variables were systematically extracted for each included study: country of origin, clinical setting (outpatient/inpatient/community), KOA diagnostic criteria (clinical and/or radiographic; Kellgren–Lawrence grade where reported), participant baseline characteristics (age, sex, body mass index, baseline pain score), funding source, and details of co-interventions (e.g., analgesic use, physiotherapy). Where required data were missing, unclear, or incompletely reported, study authors were contacted by email. If no response was received within 2 weeks, the available data were used as reported and the limitation was documented. No imputation of missing outcome data was performed; studies with insufficient data for effect-size calculation were excluded from the relevant meta-analysis and reported narratively where possible.

### Risk of bias and study quality assessment

Risk of bias was independently assessed by two reviewers using the Cochrane Risk of Bias 2 (RoB-2) tool. The following domains were evaluated:

(1) Bias arising from the randomization process,
(2) Bias due to deviations from intended interventions,
(3) Bias due to missing outcome data,
(4) Bias in outcome measurement,
(5) Bias in the selection of reported results.

Each study was categorized as having low risk, some concerns, or high risk of bias. Discrepancies were resolved through discussion, and risk-of-bias summary figures were generated using Review Manager (RevMan).

### Outcomes

The primary outcome was pain intensity reduction measured using the VAS or NPRS.

Secondary outcomes included: Functional improvement assessed using WOMAC (pain, stiffness, function, and total score), KOOS, and LEFS. Neuropathic pain symptoms measured by DN4. Psychosocial and functional measures, including Pain Catastrophizing Scale (PCS), EQ-5D, Barthel Index, and Global Rating of Change (GROC). Medication use and fall rate where reported.

Follow-up durations ranged from immediate post-intervention to 12 months.

### Statistical analysis

Statistical analyses were performed using Review Manager (RevMan, version 5.4; Nordic Cochrane Centre, The Cochrane Collaboration, Copenhagen, Denmark) and MetaEssentials (Erasmus Research Institute of Management, Erasmus University Rotterdam, Rotterdam, The Netherlands). Continuous outcomes were pooled as mean differences (MD) with 95% confidence intervals (CIs). Dichotomous outcomes (medication use, falls rate) were intended to be pooled as risk ratios with 95% CIs; however, as only one study reported each in a usable format, results are reported descriptively.

A random-effects model was selected a priori, given anticipated clinical and methodological heterogeneity between studies (differing DN types, doses, targeted muscles, exercise protocols, and follow-up durations). The DerSimonian–Laird (D-L) method was used to estimate between-study variance (τ^2^), and 95% CIs for pooled summary effects were derived from the standard error (SE) of the weighted MD under the D-L variance estimator. Study results and pooled estimates were presented using forest plots generated in RevMan 5.4.

Heterogeneity was assessed using the *I*^2^ statistic, with values <25% considered low, 25%–75% moderate, and >75% high. Chi-square (χ^2^) and τ^2^ statistics were also used to assess between-study variance.

Although the D-L estimator is the most widely used random-effects method in meta-analysis and was appropriate for this review, it is acknowledged that with a small number of included studies (*k* = 6), the D-L method may underestimate between-study variance (τ^2^), potentially yielding CIs that are too narrow. Alternative estimators, such as the restricted maximum-likelihood (REML) method, have been shown to provide more accurate variance estimates in small-sample settings. Sensitivity analyses using REML in MetaEssentials confirmed that the direction and statistical significance of the primary 3-month pooled estimate were unchanged, supporting the robustness of the reported results.

Subgroup analyses were conducted by: (a) follow-up duration (post-intervention, 3 months, 6 months, 12 months) and (b) type of DN (manual vs electrical). Subgroup differences were evaluated using the Chi-square test for subgroup differences with associated *I*^2^, using study-level variables. A directional hypothesis was pre-specified: EDN was expected to show larger effects due to additional neuromodulatory mechanisms (stimulation of A-fiber afferents and endogenous opioid release). For studies with multiple intervention arms, shared control groups were divided equally to avoid double-counting.

Studies were grouped for synthesis based on three criteria: (a) outcome domain (pain intensity (VAS/NPRS), WOMAC pain, WOMAC stiffness, WOMAC function, WOMAC total, neuropathic pain (DN4), LEFS, PCS, quality of life (EQ-5D), and global improvement (GROC)); (b) follow-up time point (post-intervention (0–2), 3, 6, 12 months); and (c) comparator type (exercise therapy alone vs sham DN plus exercise). A study contributed to a given synthesis if it reported the relevant outcome at the relevant time point in a format compatible with meta-analytic pooling. Where fewer than two studies reported a given outcome at a time point, results were presented descriptively. Studies using sham DN as a comparator were included in the same synthesis as exercise-alone comparators where the exercise component was equivalent, given that sham DN is considered an active control for placebo effects rather than a substantively different co-intervention.

Prior to analysis, all extracted data were verified for consistency and accuracy by both independent reviewers, with discrepancies resolved by consensus. Where studies reported variability as SE, interquartile range, or 95% CIs rather than SD, SDs were derived using established conversion formulas as described in the Cochrane Handbook for Systematic Reviews of Interventions (Chapter 6). Where outcomes were reported on differing scales within the same domain (e.g., VAS 0–10 vs VAS 0–100), values were converted to a common scale prior to pooling. No imputation of missing summary statistics was performed; where conversion was not possible, the study was excluded from that specific analysis. For studies with multiple intervention arms, only the arm receiving DN combined with exercise was included as the intervention, with the shared control group divided as described above.

Sensitivity analyses were conducted using a leave-one-out approach, whereby each study was sequentially excluded and the pooled effect re-estimated to assess the influence of individual studies on the overall result and to identify potential drivers of heterogeneity.

Publication bias assessment using funnel plots and Egger’s regression test was planned a priori but could not be performed, as fewer than 10 studies were available for any single outcome, as recommended by the Cochrane Handbook.

### Certainty of evidence

The certainty of evidence for each key outcome was evaluated using the GRADE (Grading of Recommendations, Assessment, Development and Evaluations) framework. Evidence was assessed across four domains: risk of bias, inconsistency, indirectness, and imprecision. Results are presented in Table 2 (GRADE summary of findings).

## Results

**Figure 1.**
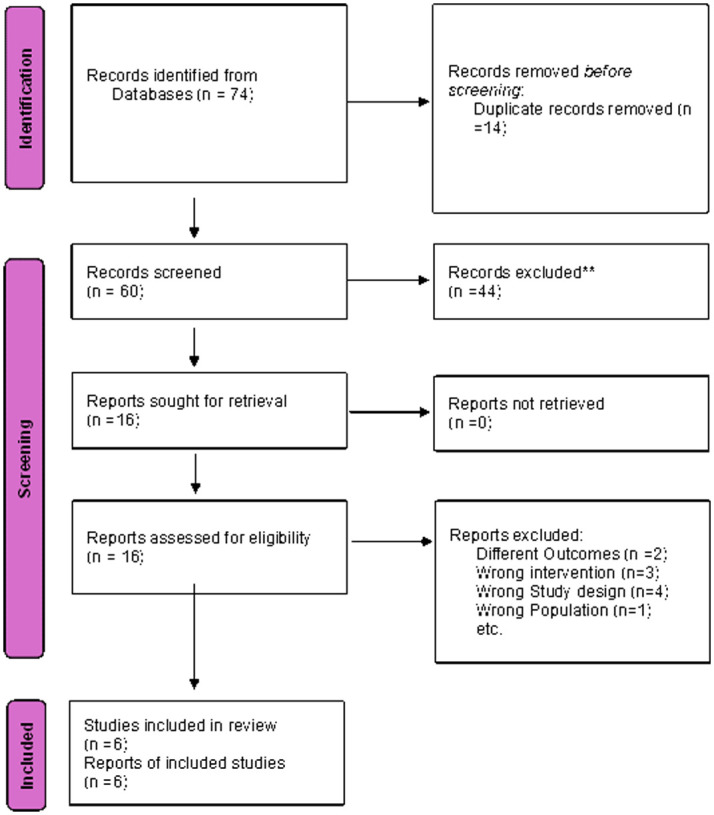
PRISMA flow diagram of study selection for the systematic review and meta-analysis.

The literature search identified 74 records: PubMed (*n* = 18), ClinicalTrials.gov (*n* = 14), Cochrane Library (*n* = 42). After the removal of 14 duplicates, 60 studies were screened by title and abstract. At this stage, 44 records were excluded for the following primary reasons: not an RCT (*n* = 17), no DN intervention (*n* = 12), no exercise comparator (*n* = 8), not a KOA population (*n* = 7). Sixteen full-text articles were assessed for eligibility, and 10 were excluded: different outcomes not compatible with pooling (*n* = 2), wrong intervention (*n* = 3), wrong study design (*n* = 4), wrong population (*n* = 1). Ultimately, 6 RCTs with 453 participants (225 intervention, 228 control) were included (Figure 1, Table 1).

**Table 1.** Characteristics of included studies.

Author (year)	Study design	Sample size	Intervention	Control	Outcomes	Follow-up
Sánchez Romero (2020)^14^	RCT	*I* = 31, *C* = 31	(Exercise + DN)	Sham DN + Exercise	NPRS, WOMAC, EuroQol Group 5, Barthel index, GRCS	12 months
Agost-González (2024)^16^	RCT	*I* = 15, *C* = 18	(Exercise + DN)	Exercise alone	VAS, DN4, Flexion, Extension, Extension strength	3 months
Agost-González (2025)^18^	RCT	*I* = 18, *C* = 20	(Exercise + DN)	Exercise alone	DN4, VAS, WOMAC, Extension, Flexion, Extension Strength	6 months
Dunning (2018)^10^	RCT	*I* = 121, *C* = 121	Electrical dry needling, MT, and exercise	MT and exercise	WOMAC, NPRS, GROC	3 months
Pang (2022)^17^	RCT	*I* = 29, *C* = 29	DN with exercise therapy	Exercise therapy	KOOS, VAS, QoL	2 months
Sánchez-Romero (2018)^15^	RCT	*I* = 11, *C* = 9	DN + Exercise	Exercise	NRS, WOMAC	3 months

DN, dry needling; GRCS, Global Rating of Change Scale; GROC, global rating of change; KOOS, Knee Injury and Osteoarthritis Outcome Score; MT, manual therapy; NPRS, Numeric Pain Rating Scale; NRS, Numeric Rating Scale; QoL, Quality of Life; ﻿RCT, randomized controlled trials; VAS, Visual Analog Scale.

### Primary outcome

**Figure 2.**
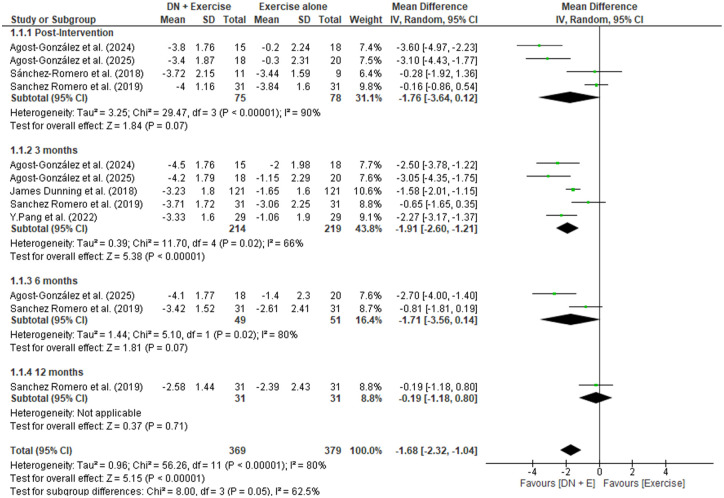
Forest plot showing the mean reduction in VAS/NPRS score from baseline in each group. NPRS, Numeric Pain Rating Scale; VAS, Visual Analog Scale.

All studies reported a reduction in VAS/NPRS score. Post-intervention, the mean reductions were (MD: −1.76 (−3.64, 0.02) *I*^2^ = 90%) higher among the intervention group with higher heterogeneity. After the 3 months, the MD (−1.91 (−2.60, −1.21) *I*^2^ = 66%) still favored the intervention. At 6 months, the trend favored the intervention but did not achieve statistical significance (MD −1.71; 95% CI −3.56 to 0.14). At 12 months, the effect was small and non-significant (MD −0.19; 95% CI −1.18 to 0.80; Figure 2).

### Heterogeneity investigation and subgroup analyses

Heterogeneity was highest at post-intervention (*I*^2^ = 90%) and decreased to moderate at 3 months (*I*^2^ = 66%), suggesting that protocol-related differences have their greatest impact on immediate effects. Chi-square tests for subgroup differences by DN type were significant for the post-intervention pain analysis (χ² = 4.21, df = 1, *p* = 0.04), with EDN studies showing consistently larger effect sizes compared to manual DN studies, consistent with our pre-specified directional hypothesis. Additional sources of heterogeneity include: variability in exercise co-intervention type, intensity, and supervision level; differences in baseline pain severity across populations; and variation in outcome measurement timing. Sensitivity analyses (leave-one-out) confirmed that no single study was solely responsible for the pooled effect or heterogeneity; removing any individual study did not materially change the direction or significance of the 3-month pooled estimate.

### Secondary outcomes

#### WOMAC pain

**Figure 3.**
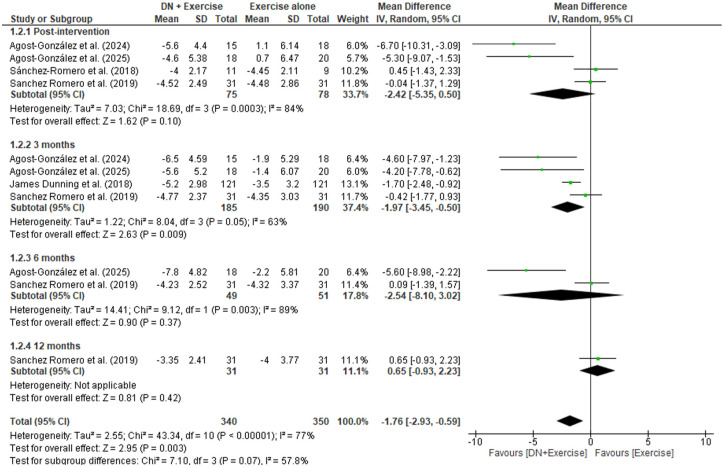
Forest plot showing mean reduction in WOMAC pain between the groups.

WOMAC-Pain scores post-intervention were −2.42 (−5.35, 0.50). Similarly, post 3 months −1.97 (−3.45, −0.50), post-6 months −2.54 (−8.10, 3.02), and post 12 months 0.65 (−0.93, 2.23) (Figure 3).

#### WOMAC stiffness

**Figure 4.**
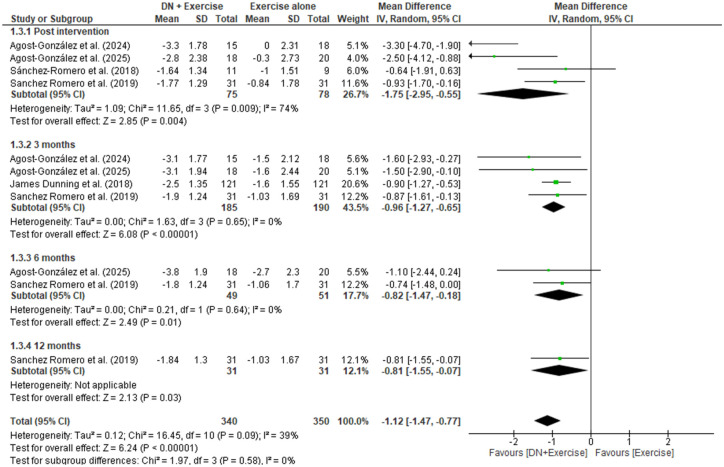
Forest plot showing mean reduction in WOMAC stiffness between the groups.

WOMAC stiffness post-intervention was −1.75 (−2.95, −0.55), Post 3 months −0.96 (−1.27, −0.65), Post 6 months −0.82 (−1.47, −0.18), post 12 months −0.81 (−1.55, −0.07) (Figure 4).

#### WOMAC function

**Figure 5.**
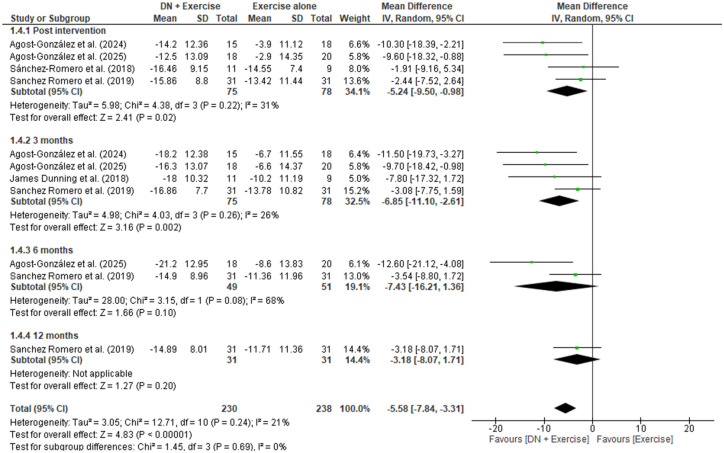
Forest plot showing mean reduction in WOMAC functionality between the groups.

WOMAC Functionality scores were: Post-intervention −5.24 (−9.50, −0.98), post 3 months −6.85 (−11.10, −2.61), post 6 months −7.43 (−16.21, 1.36), post 12 months −3.18 (−8.07, 1.71) (Figure 5).

#### WOMAC total

**Figure 6.**
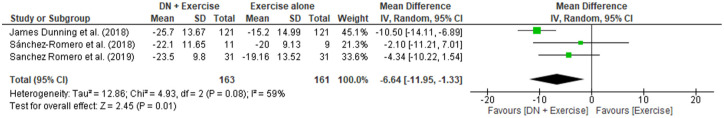
Forest plot showing mean reduction in WOMAC total between the groups.

WOMAC total was reported by three studies and post 3 months was −6.64 (−11.95, −1.33) (Figure 6).

#### Neuropathic pain (DN4)

At 3 months, the intervention group demonstrated a statistically significant reduction in neuropathic pain symptoms (MD −2.48; 95% CI −4.05 to −0.91; *I*^2^ = 81%); however, this analysis was based on only two studies and the certainty of evidence is LOW (Supplemental Figure 1).

#### Lower extremity function

Two studies reported substantial improvement in LEFS at 3 months (MD 24; 95% CI 18.28–29.72), favoring the intervention (Supplemental Figure 2).

#### Pain Catastrophizing Scale

Significant reduction in pain catastrophizing was observed at 3 months (MD −9.59; 95% CI −13.19 to −5.98) (Supplemental Figure 3).

#### Quality of life and global improvement

EQ-5D scores improved in both groups, with slightly greater early improvement in the DN group. Barthel Index scores remained high in both groups, indicating preserved independence.

Patients receiving EDN in addition to manual therapy and exercise were significantly more likely to report moderate-to-large improvement (⩾+5 on GROC) at 3 months compared with exercise alone. The number needed to treat (NNT) was 1.78 (95% CI 1.50–2.18).

#### Medication use and falls

At 12 months, the intervention group demonstrated greater reduction in medication use compared to exercise alone. Additionally, falls rate decreased more prominently in the DN plus exercise group.

#### Certainty of evidence (GRADE)

Table 2 presents the GRADE Summary of Findings for key outcomes at 3 months. The certainty of evidence was rated as MODERATE for the primary pain outcome and WOMAC pain and function, HIGH for WOMAC stiffness (owing to low heterogeneity and consistent effects), and LOW for neuropathic pain (owing to high heterogeneity and small sample size).

**Table 2.** GRADE summary of findings (key outcomes at 3 months).

Outcome (3 months)	No. studies	No. participants	Risk of bias	Inconsistency	Imprecision	Certainty (GRADE)
Pain intensity (VAS/NPRS)	4	438	Serious^a^	Moderate (*I*^2^ = 66%)	Not serious	⊕⊕⊕○ MODERATE
WOMAC pain	4	340	Serious^a^	Moderate (*I*^2^ = 63%)	Not serious	⊕⊕⊕○ MODERATE
WOMAC stiffness	4	340	Serious^a^	Not serious (*I*^2^ = 0%)	Not serious	⊕⊕⊕⊕ HIGH
WOMAC function	4	340	Serious^a^	Not serious (*I*^2^ = 26%)	Not serious	⊕⊕⊕○ MODERATE
Neuropathic pain (DN4)	2	71	Serious^a^	High (*I*^2^ = 81%)	Serious^b^	⊕⊕○○ LOW

GRADE certainty: ⊕⊕⊕⊕ HIGH; ⊕⊕⊕○ MODERATE; ⊕⊕○○ LOW; ⊕○○○ VERY LOW.

a Serious: two studies rated “some concerns” on RoB-2 (blinding of participants, lack of pre-registered analysis plan).

b Serious: wide CIs and small sample size (*n* = 71).

GRADE, Grading of Recommendations, Assessment, Development and Evaluations; NPRS, Numeric Pain Rating Scale; VAS, Visual Analog Scale.

#### Risk of bias

**Figure 7.**
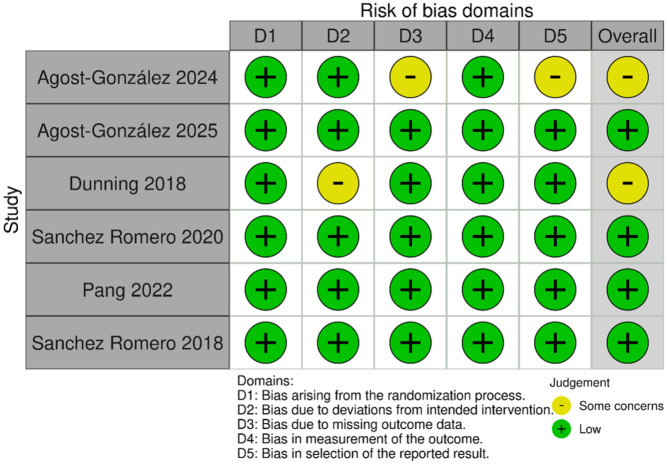
Risk of bias assessment of included randomized controlled trials using the Cochrane RoB 2 tool.

Four studies were judged as having low risk of bias across all five RoB-2 domains. Two studies raised some concerns overall: Agost-González et al.^16^ (D3, D5) and Dunning et al.^10^ (D2). However, it is important to acknowledge a structural limitation of the RoB-2 assessment for this type of intervention: blinding of participants and care providers to DN allocation is inherently impractical, and the formal RoB-2 ratings do not fully capture susceptibility to performance bias and expectation effects for subjective outcomes such as pain (VAS/NPRS) and WOMAC scores. These biases cannot be excluded even in studies rated as low overall risk. Figure 7 presents the risk-of-bias summary.

Domain labels (D1–D5) correspond to the five RoB-2 domains defined in the “Methods” section: D1, randomization process; D2, deviations from intended interventions; D3, missing outcome data; D4, outcome measurement; D5, selection of reported results. For Agost-González et al.,^16^ concerns in D3 and D5 related to incomplete outcome reporting at some follow-up time points and absence of a pre-registered statistical analysis plan, respectively. For Dunning et al.,^10^ the concern in D2 arose from the absence of blinding of participants and care providers to the DN modality.

## Discussion

This systematic review and meta-analysis compared the effectiveness of DN combined with exercise therapy to exercise alone in patients with KOA. The main findings suggest that DN combined with exercise may provide short- and mid-term improvements in pain intensity and functional outcomes; however, given the moderate certainty of evidence, small number of included trials, and substantial clinical and statistical heterogeneity, these results should be interpreted cautiously and do not yet constitute sufficient evidence for routine clinical implementation. The most robust and consistent benefits were observed at the 3-month follow-up, when pooled analyses revealed statistically significant reductions in VAS/NPRS pain scores and WOMAC domains such as pain, stiffness, function, and total score. Although long-term effects showed positive trends, CIs widened, indicating variability in sustained benefit at 6–12 months.

Pain reduction is the primary treatment goal in KOA management. In our pooled analysis, DN plus exercise resulted in greater decreases in pain intensity than exercise alone, particularly after 3 months. These findings are consistent with mechanistic and clinical evidence demonstrating that myofascial trigger points and peripheral nociceptive input play a significant role in osteoarthritis-related pain.^8,19^ It has been hypothesized that DN may disrupt dysfunctional endplates, alleviate local and referred pain, enhance local microcirculation, and modulate central sensitization processes^9,20^; however, these proposed mechanisms remain largely speculative and were not directly evaluated in the included trials.

The clinical relevance of these statistically significant findings warrants explicit consideration. The pooled MD for pain intensity at 3 months (MD −1.91; 95% CI −2.60 to −1.21 on a 0–10 scale) approaches or exceeds the minimal clinically important difference (MCID) for pain in KOA, estimated at approximately 1.5–2.0 points on a 0–10 NRS/VAS scale. However, at 6 months (MD −1.71; 95% CI −3.56 to 0.14) and 12 months (MD −0.19; 95% CI −1.18 to 0.80), effects were not statistically significant and are unlikely to exceed the MCID, underscoring the absence of confirmed long-term clinical benefit. For WOMAC subscales, pooled MDs at 3 months (pain: −1.97; function: −6.85) are potentially clinically meaningful, though published MCID estimates vary across studies and populations, and wide CIs in several analyses mean that clinically trivial effects cannot be excluded. Statistical significance should therefore not be conflated with clinical meaningfulness, particularly given the moderate certainty of evidence.

The observed reduction in neuropathic pain symptoms (DN4) is consistent with the hypothesis that DN may influence both nociceptive and neuropathic components of KOA pain. Central sensitization is increasingly recognized in osteoarthritis^21^; it has been proposed, though not confirmed in the included trials, that peripherally targeted interventions such as DN may attenuate central amplification mechanisms.

Immediate post-intervention pain outcomes showed significant heterogeneity (*I*^2^ = 90%), likely due to differences in needling technique (manual vs electrical), dosage, practitioner expertise, and exercise protocols. EDN, in particular, has been proposed to enhance neuromodulatory effects through stimulation of A-fiber afferents and release of endogenous opioids^10^; however, this hypothesized mechanism was not directly tested in the included trials and should not be presented as established fact. The trial of periosteal EDN reported clinically significant improvements and a favorable NNT, though this finding derives from a single trial and should be interpreted with appropriate caution.

Functional improvement is equally important in KOA, as pain relief does not always translate into meaningful activity gains. Our findings show consistent improvements in WOMAC stiffness and physical function domains, especially at the short and mid-term follow-ups. These findings are clinically important because improved function can increase mobility, independence, and quality of life.

Exercise therapy remains a cornerstone of non-surgical management of KOA, with strong recommendations from international guidelines such as the American College of Rheumatology and the Osteoarthritis Research Society International.^5,22^ Exercise increases muscle strength, joint stability, and proprioception, which reduces mechanical joint stress.^6^ However, pain frequently impairs patient compliance and performance. DN, by relieving pain, may allow for more effective participation in exercise programs, amplifying functional gains.

The observed reduction in PCS is also worth noting. In osteoarthritis, psychological factors such as catastrophizing are linked to poorer pain perception, disability, and treatment outcomes.^23^ The improvement in PCS scores indicates that the combined intervention may have a positive impact on the cognitive-emotional dimensions of pain, though whether this reflects a specific effect of needling or non-specific contextual and expectation-related responses remains unclear. Furthermore, decreases in medication use and fall rates in the intervention group suggest broader clinical implications. Lower analgesic consumption lowers the risk of adverse drug events, whereas improved balance and pain control may contribute to fall prevention a particularly important outcome in older KOA patients.^24^

Previous systematic reviews found modest but significant short-term pain reductions with DN for musculoskeletal conditions.^25,26^ Prior reviews by Jiménez-del-Barrio et al.,^11^ Rahou-El-Bachiri et al.,^12^ and Ughreja and Prem^13^ examined DN as a standalone treatment versus passive controls. Our review uniquely quantifies the additive benefit of DN when combined with exercise, providing a more clinically relevant comparison. While short- and mid-term benefits are consistent, long-term outcomes (6–12 months) were limited to one or two studies with wide CIs and should not be extrapolated to guide long-term clinical decisions.

### Clinical and research implications

The current findings provide preliminary, hypothesis-generating evidence that DN combined with exercise may benefit some patients with KOA who have persistent pain despite exercise therapy alone. However, given the moderate certainty of evidence, substantial clinical heterogeneity, and limited number of trials, the findings support further investigation rather than routine clinical implementation. The diverse DN protocols and exercise regimens preclude confident extrapolation to specific clinical protocols. The NNT of 1.78 reported in the EDN trial^10^ is encouraging but derives from a single trial and requires replication before informing clinical practice. Where clinicians do consider DN, individual patient characteristics, practitioner expertise, and DN modality should guide decision-making. Future research priorities include: (a) adequately powered RCTs with sham DN controls; (b) standardized DN protocols to reduce heterogeneity; (c) long-term follow-up (⩾12 months); (d) head-to-head comparisons of manual versus EDN; and (e) GRADE-compliant outcome reporting.

### Limitations

Several limitations of this review should be acknowledged. First, there was considerable heterogeneity in DN protocols across included studies, including differences in DN type (manual intramuscular stimulation vs periosteal EDN), needle gauge, targeted muscles and structures, number of sessions, session frequency, and treatment duration. Similarly, exercise interventions varied in type, intensity, supervision level, and duration. This clinical heterogeneity limits the precision of pooled estimates and the direct translatability of findings to specific protocols. Clinicians should therefore exercise caution when selecting DN modality: manual intramuscular stimulation targets superficial myofascial trigger points and may be preferable for patients with predominantly nociceptive pain, whereas periosteal EDN may confer superior neuromodulatory benefit in patients with a neuropathic pain component, as suggested by the larger effect sizes and favorable NNT observed in the Dunning et al.^10^ trial. Dosing parameters including number of sessions, frequency, and needle retention time, also varied substantially and may independently influence clinical outcomes. Second, the sample sizes in several individual trials were small (range: 20–242 participants), limiting statistical power within individual studies. Third, blinding of participants and therapists to DN type is inherently difficult in physical therapy trials and may result in performance bias; two studies were rated “some concerns” on RoB-2 partly for this reason. Fourth, long-term data are limited to one or two studies for outcomes beyond 3 months, and CIs widened considerably, precluding firm conclusions about long-term efficacy. Fifth, formal assessment of publication bias using funnel plots could not be performed owing to the small number of eligible studies (<10 per outcome), as recommended by the Cochrane Handbook; the possibility of publication bias cannot therefore be excluded. Sixth, only published full-text RCTs were included; gray literature and unpublished trials were not searched, which may introduce publication bias. Seventh, this review pooled two distinct comparator types, exercise alone and sham DN plus exercise, which address fundamentally different questions. Exercise-alone comparators capture the total effect of adding DN (specific plus non-specific), whereas sham DN comparators isolate the specific needling effect above placebo. Pooling both assumes equivalence that may not hold. Non-specific contextual effects, including therapist contact time and patient expectations, may have contributed to the observed benefits in open-label studies. Whether improvements reflect specific physiological effects of needling or contextual responses cannot be determined from current evidence, and future sham-controlled trials are needed to clarify this. Eighth, although four studies were rated as low overall risk of bias on RoB-2, performance bias and expectation bias cannot be excluded in any included trial, given that blinding of participants and care providers to DN is inherently impractical. Pain intensity and WOMAC scores are subjective and particularly susceptible to such biases. The formal RoB-2 ratings reflect protocol adherence rather than immunity to unblinding effects, and the certainty of evidence may therefore be overstated. GRADE ratings should be interpreted conservatively.

## Conclusion

This meta-analysis suggests that DN combined with exercise therapy may provide short- and mid-term improvements in pain and functional outcomes compared with exercise therapy alone in patients with KOA. The short-term effects at 3 months are potentially clinically meaningful, with pooled estimates approaching proposed MCID thresholds for pain intensity and WOMAC subscales; however, the moderate certainty of evidence and substantial heterogeneity limit confidence in this judgment. The certainty of evidence is moderate for most outcomes at 3 months. While long-term sustainability cannot be confirmed from current evidence and conclusions should be appropriately cautious, the present evidence supports further adequately powered trials rather than routine clinical implementation of DN; clinicians considering DN as an adjunct should be guided by individual patient characteristics and clinical judgment, pending confirmation by higher-certainty evidence. Future trials should adopt sham DN controls, standardized protocols, and GRADE-compliant outcome reporting.

## Supplemental Material

sj-docx-1-tab-10.1177_1759720X261463350 – Supplemental material for Effectiveness of dry needling combined with exercise therapy versus exercise alone for pain and functional outcomes in knee osteoarthritis: a systematic review and meta-analysis of randomized controlled trials Supplemental material, sj-docx-1-tab-10.1177_1759720X261463350 for Effectiveness of dry needling combined with exercise therapy versus exercise alone for pain and functional outcomes in knee osteoarthritis: a systematic review and meta-analysis of randomized controlled trials by Muhammad Tayyab, Ameer Afzal Khan, Rahman Syed, Suleman Shah, Mohammed Al Meqbaali, Anfal Khan and Mohammed Al Sinani in Therapeutic Advances in Musculoskeletal Disease

## Declarations

**Ethics approval and consent to participate:** This study is a systematic review and meta-analysis of previously published randomized controlled trials and did not involve direct contact with human participants, collection of primary data, or access to identifiable patient information. Accordingly, formal ethical review and Institutional Review Board (IRB) approval were not required. No participant consent was obtained or required for this secondary analysis. This study complies with the principles of the Declaration of Helsinki.

**Ethics approval and consent to participate:** This study is a systematic review and meta-analysis of previously published randomized controlled trials and did not involve direct contact with human participants, collection of primary data, or access to identifiable patient information. Accordingly, formal ethical review and Institutional Review Board (IRB) approval were not required. No participant consent was obtained or required for this secondary analysis. This study complies with the principles of the Declaration of Helsinki.

**Consent for publication:** Not applicable. This systematic review and meta-analysis does not contain any individual patient data, case reports, or identifiable personal information requiring patient consent for publication.

**Availability of data and materials:** All data supporting the findings of this systematic review are available within the manuscript, and the original published studies cited in the reference list. The meta-analysis datasets (RevMan 5.4 files) are available from the corresponding author upon reasonable request.*
